# Green Fluorescent Protein- and *Discosoma* sp. Red Fluorescent Protein-Tagged Organelle Marker Lines for Protein Subcellular Localization in Rice

**DOI:** 10.3389/fpls.2019.01421

**Published:** 2019-11-05

**Authors:** Ziqiang Chen, Wenhui Zheng, Longhai Chen, Chenlu Li, Tingmin Liang, Zaijie Chen, Huibing Xu, Yijuan Han, Lan Kong, Xu Zhao, Feng Wang, Zonghua Wang, Songbiao Chen

**Affiliations:** ^1^Biotechnology Research Institute, Fujian Academy of Agricultural Sciences, Fuzhou, China; ^2^State Key Laboratory of Ecological Pest Control for Fujian and Taiwan Crops, College of Plant Protection, Fujian Agriculture and Forestry University, Fuzhou, China; ^3^Temasek Life Sciences Laboratory, and the Department of Biological Sciences, National University of Singapore, Singapore, Singapore; ^4^Marine and Agricultural Biotechnology Laboratory, Institute of Oceanography, Minjiang University, Fuzhou, China

**Keywords:** fluorescent protein, organelle marker lines, protein subcellular localization, rice, *Magnaporthe oryzae*

## Abstract

The subcellular localization of proteins is a fundamental aspect of protein functions. Determining the subcellular localization is important for understanding the biological functions of proteins. Here, we developed a set of rice organelle marker lines, in which the expressing fluorescent organelle markers could be used as comparative standards in determining the subcellular localization of the protein of interest. We constructed green fluorescent protein (GFP)- and/or *Discosoma* sp. red fluorescent protein (DsRed)-tagged organelle markers targeted to the endoplasmic reticulum (ER), mitochondria, Golgi apparatus, peroxisome, actin cytoskeleton, plastid, tonoplast, plasma membrane, and nucleus, respectively. The utility of the rice marker lines for protein subcellular localization studies was demonstrated by detecting a nucleus-localized OsWRKY45 and a mitochondria-associated NbHxk1 in protoplasts of the GFP-OsH2B and the ScCOX4-DsRed lines, respectively. Using a sheath-inoculation method, followed by a live-cell imaging, we detected co-localization of a *Magnaporthe oryzae* PWL2:mCherry : NLS fusion with the nucleus marker in the GFP-OsH2B rice epidermal cells, confirming the translocation of the *M. oryzae* effector PWL2 into host cells, and further demonstrating the feasibility of using the organelle marker lines for studying dynamics of proteins in rice cells in the interactions between rice and pathogens. The set of organelle marker lines developed in the present study, provides a valuable resource for protein subcellular localization studies in rice.

## Introduction

Eukaryotic cells are highly compartmentalized and various biological processes are implemented within specialized subcellular compartments (Nelson et al., 2007). In general, the subcellular localization of proteins is closely associated with functions related to the biological processes carried out in the corresponding organelles (Lunn, 2007; Mathur, 2007). Thus, information on the subcellular localization is helpful in understanding the biological functions of proteins (Martin et al., 2009; Tanz et al., 2013).

Protein subcellular localization analysis has been routinely employed as an experimental methodology for gene functional studies. Over the past decades, fluorescent protein (FP) tagging has become the dominant approach for determining the subcellular localization of proteins in living cells in different organisms, including plants (Chudakov et al., 2010; Tanz et al., 2013). Based on fusion of FPs with the well-established targeting proteins or motifs (Mathur, 2007), numerous sets of fluorescent organelle markers have been developed for *Arabidopsis* (Nelson et al., 2007; Geldner et al., 2009; Kim et al., 2013), *Nicotiana benthamiana* (Martin et al., 2009), *Medicago truncatula* (Luo and Nakata, 2012), maize (Wu et al., 2013; Krishnakumar et al., 2015), and rice (Wu et al., 2016; Dangol et al., 2017). Currently, the common strategy utilized to detect localization of FP-tagged proteins has been based on transient expression of fusion constructs in protoplasts through polyethylene glycol (PEG)-mediated transfection (Chen et al., 2006; Zhang et al., 2011), or in epidermal cells through biolistic bombardment (Dangol et al., 2017) or agro-infiltration (Lunn, 2007; Nelson et al., 2007). Although transient expression systems provide convenient and rapid assays for subcellular localization, the approaches have limitations in monitoring processes in a developmental context or under particular environmental conditions, such as in response to abiotic stresses, or pathogen attack (Geldner et al., 2009). Recently, transgenic lines stably expressing FP-tagged organelle markers have been generated in *Arabidopsis*, *M. truncatula*, maize, and rice (Geldner et al., 2009; Luo and Nakata, 2012; Kim et al., 2013; Wu et al., 2013; Krishnakumar et al., 2015; Wu et al., 2016). These transgenic marker lines provided valuable resources for protein subcellular localization studies, especially for studying the dynamics of proteins or organelles in developmental or environmental contexts (Geldner et al., 2009; Kim et al., 2013; Wu et al., 2016).

Rice is one of the most important food crops in the world, as well as a monocot model plant. More recently, sets of fluorescent organelle markers have been developed for protein subcellular localization studies in rice (Wu et al., 2016; Dangol et al., 2017). However, only green fluorescent protein (GFP)-based organelle marker lines have been generated in rice (Wu et al., 2016). In the present study, to provide more flexible combinations of organelle marker lines, we developed transgenic rice plants stably expressing GFP- and/or *Discosoma* sp. red fluorescent protein (DsRed)-tagged organelle markers. We demonstrated the utility of the marker lines for co-localization studies by detecting a nucleus-localized rice OsWRKY45 and a mitochondria-associated *N. benthamiana* hexokinase NbHxk1 in protoplasts of the GFP-OsH2B and the ScCOX4-DsRed rice lines, respectively. In addition, we demonstrated the feasibility of using the organelle marker lines for studying dynamics of proteins in rice cells in the interactions between rice and pathogens. Using a sheath-inoculation method, followed by a live-cell imaging (Khang et al., 2010; Park et al., 2012), we detected co-localization of a *Magnaporthe oryzae* PWL2:mCherry : NLS fusion with the nucleus marker in the GFP-OsH2B rice epidermal cells, confirming the translocation of the *M. oryzae* effector PWL2 into host cells. The organelle markers and marker lines developed in this study, along with previously developed organelle markers and marker lines (Wu et al., 2016; Dangol et al., 2017), therefore, provide valuable resources for protein localization studies in rice.

## Materials and Methods

### Generation of Organelle Marker Constructs

The s*GFP*(S65T) gene (Chiu et al., 1996) and the *DsRed* gene (Goodin et al., 2002) were used for the construction of fluorescent organelle marker constructs. *GFP*- or *DsRed*-fusion fragments were generated by overlapping PCR and were cloned into a plant binary vector, pCXSN (Chen et al., 2009) in which the *GFP*- or *DsRed*-fusions were driven by the CaMV 35S promoter. All constructs were confirmed by sequencing. Primers used for PCR or overlapping PCR were listed in **Supplementary Table S1**.

### Agro-Infiltration Assay in *Nicotiana benthamiana*

The organelle marker constructs were introduced into the *Agrobacterium tumefaciens* strain GV3101. Transformed GV3101 bacteria were cultured in liquid yeast extract peptone media supplemented with kanamycin (50 ug/ml) and rifampicin (50 ug/ml). Suspensions of transformed GV3101 bacteria were adjusted to an OD_600_ of 0.7 in the infiltration buffer (10 mM MES, 10 mM MgCl_2_, and 150 µM acetosyringone), and were maintained at room temperature for 2 h. The suspension cultures were infiltrated into leaves of 4-week-old *N. benthamiana* plants grown in a growth chamber at 25°C under 16/8 h light/dark cycle.

### Rice Transformation

The organelle marker constructs were introduced into *A. tumefaciens* LBA4404 by electroporation. Rice calli induced from the embryos of mature seeds of cv. Nipponbare were used for transformation *via* the *Agrobacterium*-mediated method as described previously (Hiei et al., 1994). Regenerated transgenic plants and their self-pollinated progeny were grown in a greenhouse.

### Rice Protoplast Isolation and Transfection

Protoplasts were isolated from 2-week-old rice seedlings of transgenic rice organelle marker lines or wild type rice cv. Nipponbare. PEG-mediated protoplast transfection was performed as described previously (Chen et al., 2006). Protoplasts were incubated under dark at room temperature for 16–24 h.

### Rice Leaf Sheath Inoculation With *Magnaporthe oryzae*

A transgenic *M. oryzae* isolate carrying a *PWL2:mCherry : NLS* expression cassette (Khang et al., 2010) was used for rice sheath inoculation. Rice leaf sheath inoculation with *M. oryzae* was performed as described previously (Khang et al., 2010; Park et al., 2012). In brief, leaf sheaths were detached from 4-week-old rice seedlings, and were cut into 5–6 cm segments. Inoculation was performed by incubating fungal spores (1 × 10^5^ conidial/ml) in inner interior of leaf sheaths. The inoculated sheaths were placed in a petri dish and were incubated under high humidity (95%) and at 26°C for about 2 days before microscopic inspection.

### Fluorescence Visualization

Fluorescence imaging was performed on a Leica DMi8 Laser Scanning Confocal microscope (Leica, Wetzlar, Germany). Excitation/emission wavelengths were 488/535 nm for GFP, and 552/610 nm for RFP.

## Results

### Plant Expression Cassettes for Green Fluorescent Protein –/*Discosoma* sp. Red Fluorescent Protein-Fusion Organelle Markers

**Figure 1:**
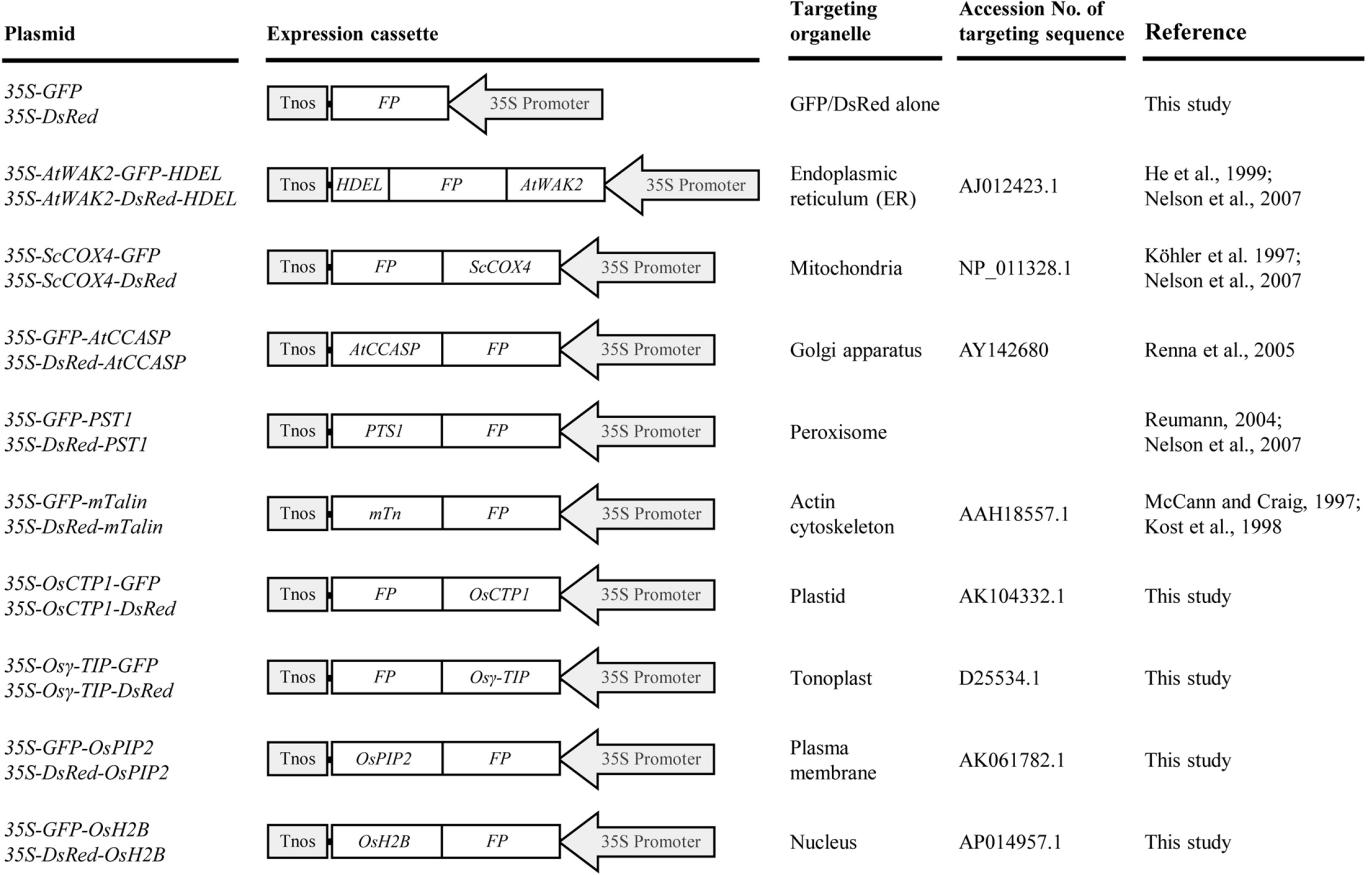
List of green fluorescent protein (GFP)- and *Discosoma* sp. red fluorescent protein (DsRed)-tagged organelle markers developed in this study. All chimeric *GFP* or *DsRed* fusion genes were placed under the control of a CaMV 35S promoter. 35S Promoter, CaMV 35S promoter; *FP*, fluorescent protein gene *GFP* or *DsRed*; *AtWAK2*, signal peptide sequence of *AtWAK2* gene; *HDEL*, ER retention signal sequence; *ScCOX4*, transit sequence of *ScCOX4*; *AtCCASP*, coding sequence of C-terminal region of AtCASP; *PTS1*, peroxisomes targeting signal 1 sequence; *mTn*, coding sequence of C-terminal residues of mouse mTalin; *OsCTP1*, transit peptide sequence of rice rubisco activase small subunit; *Osγ-TIP*, coding sequence of rice vacuolar membrane aquaporin; *OsPIP2*, coding sequence of rice plasma membrane aquaporin; *OsH2B*, coding sequence of rice histone 2B; Tnos, nopaline synthase terminator.

To develop rice marker lines with flexibility for co-localization assays, two commonly used FP genes, *GFP* and *DsRed*, were used to generate plant expression cassettes for organelle markers. The fusion markers were designed to target the endoplasmic reticulum (ER), mitochondria, Golgi apparatus, peroxisome, actin cytoskeleton, plastid, tonoplast, plasma membrane, and nucleus, respectively (**Figure 1**). The targeting signal sequences for the ER [signal peptide of AtWAK2 (He et al., 1999; Nelson et al., 2007) at the N-terminus and a His-Asp-Glu-Leu (HDEL) motif at the C-terminus of FPs], mitochondria (first 29-AA transit peptide of ScCOX4) (Köhler et al., 1997; Nelson et al., 2007), Golgi apparatus (C-terminal 125-AA residues of AtCASP) (Renna et al., 2005), peroxisomes [peroxisomes targeting signal 1 (PTS1), Ser-Lys-Leu motif] (Reumann, 2004; Nelson et al., 2007), and actin cytoskeleton [C-terminal residues (AA 2345-2541) of mouse mTalin] (McCann and Craig, 1997; Kost et al., 1998) were achieved according to previous reports. As for developing markers targeted to the plastid, tonoplast, plasma membrane, and nucleus, the coding sequences of rice orthologs of the first 48-AA transit peptide of rubisco activase small subunit (OsCTP1), the vacuolar membrane aquaporin (Osγ-TIP), the plasma membrane aquaporin (OsPIP2), and the histone 2B (OsH2B) were fused with *GFP* or *DsRed*, respectively (**Figure 1**). The *GFP*- or *DsRed*-fusions were placed under the control of the CaMV 35S promoter, allowing for high-level expression in both dicot and monocot plants (Chen et al., 2009).

### Subcellular Localizations of Rice OsCTP1, Osγ-TIP, OsPIP2, and OsH2B in *Nicotiana benthamiana*

**Figure 2:**
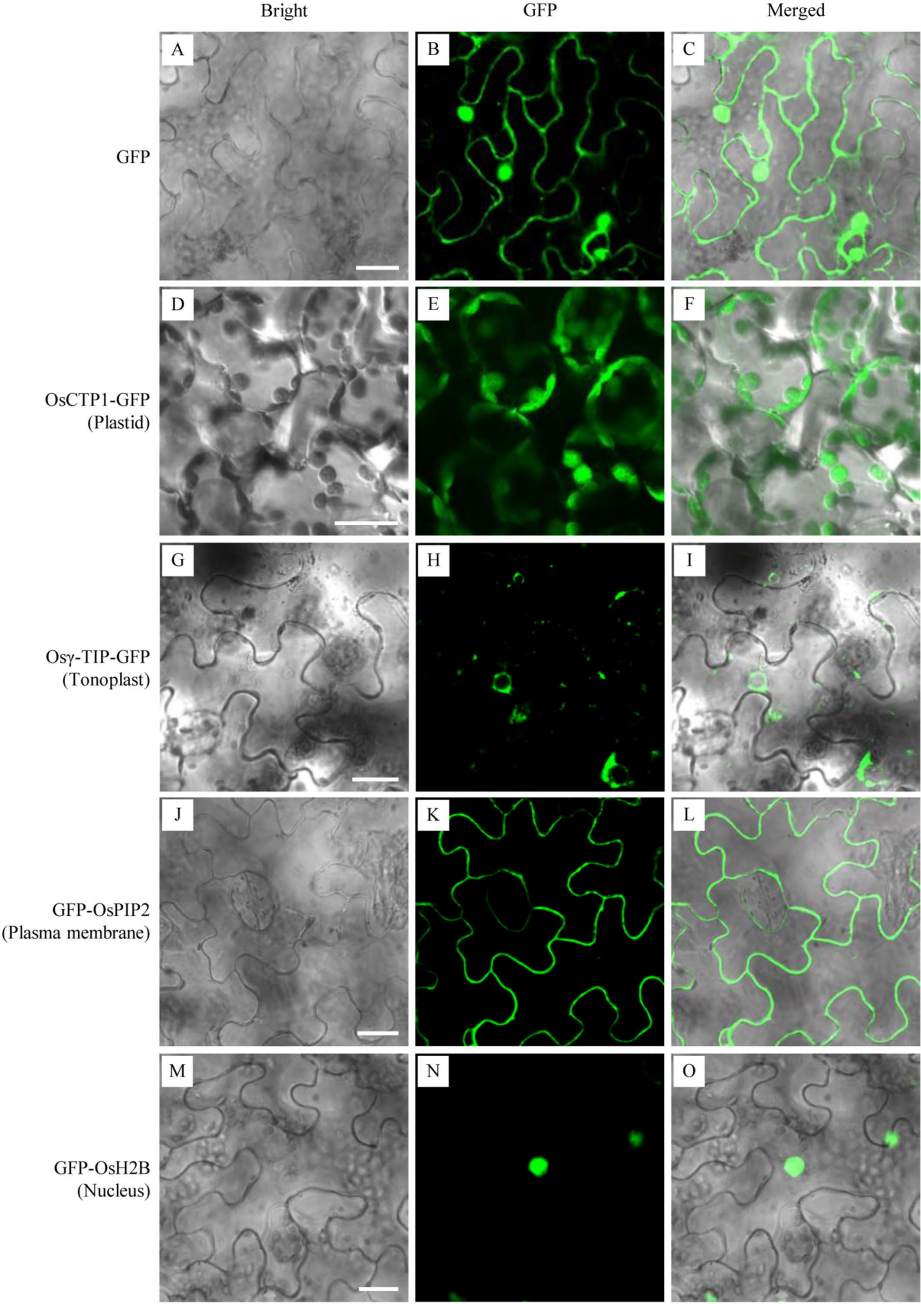
Transient validation of green fluorescent protein (GFP) fusions of four rice orthologs. The binary vectors harboring expression cassettes of GFP, OsCTP1-GFP, Osγ-TIP-GFP, GFP-OsPIP2, and GFP-OsH2B were transiently expressed in N. benthamiana epidermal cells, respectively. The subcellular localizations of GFP alone throughout the whole cells **(A**–**C)**, OsCTP1-GFP at the granular plastids **(D**–**F)**, Osγ-TIP-GFP in the shape of small rings surrounds the vacuole **(G**–**I)**, GFP-OsPIP2 at the periphery of the cells **(J**–**L)**, and GFP-OsH2B targeted to the nucleus **(M**–**O)** were shown. Scale bars, 10 μm.

To validate the subcellular localization patterns of the four rice ortholog organelle markers, GFP-fusions of rice OsCTP1, Osγ-TIP, OsPIP2, and OsH2B were transiently expressed and investigated in *N. benthamiana* leaves, respectively. Microscopy of leaf epidermal cells showed that, while *N. benthamiana* leaves infiltrated with the *35S-GFP* expression cassette displayed green fluorescence distributed uniformly throughout the whole cells (**Figures 2B, C**), the expression of OsCTP1-GFP, Osγ-TIP-GFP, GFP-OsPIP2, or GFP-OsH2B showed specific fluorescence patterns as expected: green fluorescence emitted from OsCTP1-GFP accumulated at the granular plastids (**Figures 2E, F**); Osγ-TIP-GFP signal appeared in the shape of small rings surrounds the vacuole (**Figures 2H, I**); fluorescent signal of GFP-OsPIP2 located at the periphery of the cells (**Figures 2K, L**); and GFP-OsH2B produced green fluorescence targeted to the nucleus of the epidermal cells (**Figures 2N, O**). These results also suggested that the four rice orthologs, OsCTP1, Osγ-TIP, OsPIP2, and OsH2B had conserved subcellular localization patterns across dicot and monocot species.

### Generation of Stable Rice Lines Expressing Green Fluorescent Protein/*Discosoma* sp. Red Fluorescent Protein-Fusion Organelle Markers

The binary vectors harboring expression cassettes for GFP/DsRed-fusion organelle markers were introduced into the rice cultivar Nipponbare by *Agrobacterium*-mediated transformation, and more than 20 independent T_0_ plants were generated for each construct. T_0_ plants were screened by detecting fluorescent signals from the sliced-sheath tissues, and T_1_ lines derived from T_0_ plants with good fluorescent signal were used for detailed analyses.

**Figure 3:**
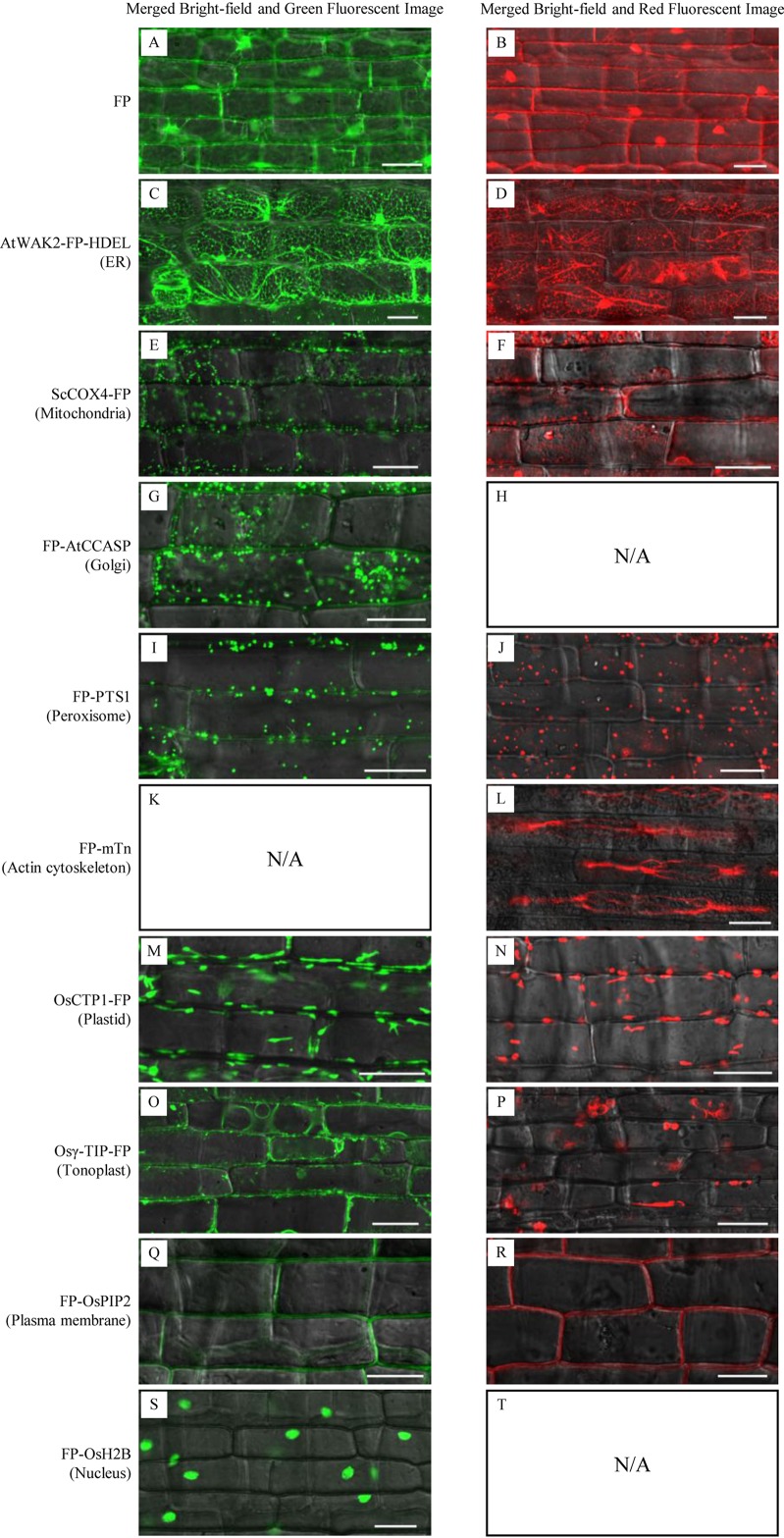
Subcellular localization of green fluorescent protein (GFP)/Discosoma sp. red fluorescent protein (DsRed)-fusion organelle markers in epidermal cells of stable transgenic rice plants. FP, fluorescent protein GFP or DsRed. Sheath epidermal cells of GFP-fusion (left panel) and DsRed-fusion (right panel) transgenic lines were examined by confocal microscopy. **(A**, **B)** GFP or DsRed alone; **(C**, **D)** ER marker AtWAK2-GFP-HDEL or AtWAK2-DsRed-HDEL; **(E**, **F)** Mitochondrial marker ScCOX4-GFP or ScCOX4-DsRed; **(G)** Golgi marker GFP-AtCCASP; **(I**, **J)** Peroxisome marker GFP-PTS1 or DsRed-PTS1; **(L)** cytoskeleton marker DsRed-mTn; **(M**, **N)** Plasmid marker CTP1-GFP or CTP1-DsRed; **(O**, **P)** Tonoplast marker Osγ-TIP-GFP or Osγ-TIP-DsRed; **(Q**, **R)** Plasma membrane marker GFP-OsPIP2 or DsRed-OsPIP2; **(S)** Nucleus marker GFP-OsH2B. N/A, No DsRed-AtCCASP- **(H)**, GFP-mTn- **(K)**, or DsRed-OsH2B-transgenic rice plants expressing obvious fluorescent signals **(T)** were obtained. Scale bars, 25 μm.

**Figure 4:**
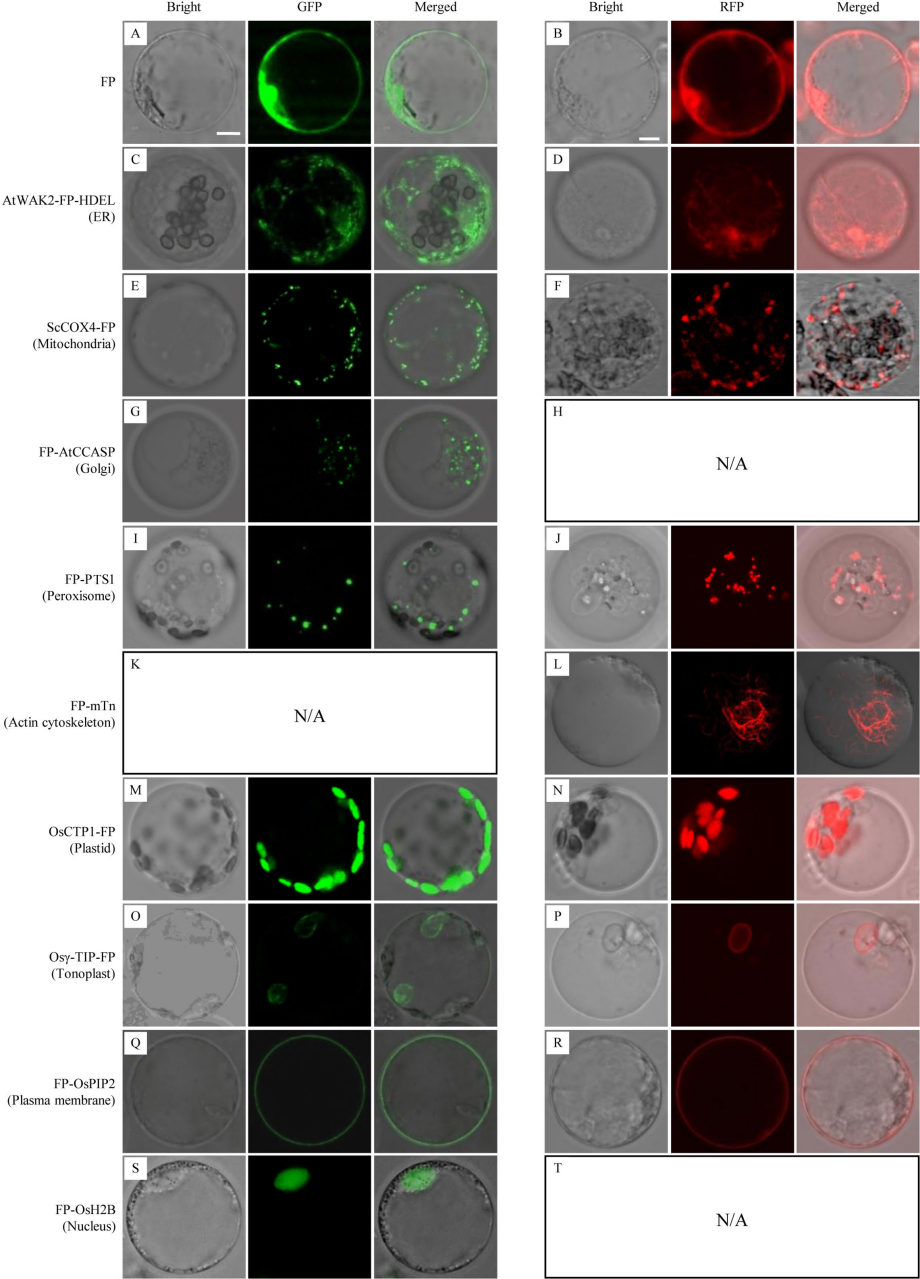
Subcellular localization of green fluorescent protein (GFP)/Discosoma sp. red fluorescent protein (DsRed)-fusion organelle markers in protoplasts of stable transgenic rice plants. FP, fluorescent protein GFP or DsRed. Protoplasts of GFP-fusion (left panel) and DsRed-fusion (right panel) transgenic lines were examined by confocal microscopy. **(A**, **B)** GFP or DsRed alone; **(C**, **D)** ER marker AtWAK2-GFP-HDEL or AtWAK2-DsRed-HDEL; **(E**, **F)** Mitochondrial marker ScCOX4-GFP or ScCOX4-DsRed; **(G)** Golgi marker GFP-AtCCASP; **(I**, **J)** Peroxisome marker GFP-PTS1 or DsRed-PTS1; **(L)** cytoskeleton marker DsRed-mTn; **(M**, **N)** Plasmid marker CTP1-GFP or CTP1-DsRed; **(O**, **P)** Tonoplast marker Osγ-TIP-GFP or Osγ-TIP-DsRed; **(Q**, **R)** Plasma membrane marker GFP-OsPIP2 or DsRed-OsPIP2; **(S)** Nucleus marker GFP-OsH2B. N/A, No DsRed-AtCCASP- **(H),** GFP-mTn- **(K)**, or DsRed-OsH2B-transgenic rice plants expressing obvious fluorescent signals **(T)** were obtained. Scale bars, 10 μm.

Microscopic analysis of sheath cells showed that most of the GFP/DsRed-fusions could be stably expressed in rice (**Figure 3**). Fluorescent signals in transgenic rice expressing AtWAK2-GFP-HDEL or AtWAK2-DsRed-HDEL exhibited typical ER localization patterns, forming reticulate networks throughout the cytoplasm (**Figures 3C, D**). The mitochondria targeting ScCOX4-GFP or ScCOX4-DsRed appeared as small spots, randomly distributed in the cytoplasm (**Figures 3E, F**). GFP-AtCCASP showed punctate fluorescence corresponding to the typical Golgi localization pattern (**Figure 3G**). GFP or DsRed fused with the C-terminal peroxisome-tagging PTS1 motif, Ser-Lys-Leu, both appeared as spherical dots dispersed in the cytosol (**Figures 3I, J**). Red fluorescence labeling of the actin cytoskeleton was observed in *DsRed-mTn*-transgenic rice lines, showing as a dense filamentous network (**Figure 3L**). The fluorescence patterns of OsCTP1-GFP (**Figure 3M**), Osγ-TIP-GFP (**Figure 3O**), GFP-OsPIP2 (**Figure 3Q**), and GFP-OsH2B (**Figure 3S**) in transgenic rice were consistent, respectively, with the plastid-, tonoplast-, plasma membrane-, and nucleus-localization patterns observed in *N. benthamiana*. The OsCTP1-DsRed, Osγ-TIP-DsRed, and DsRed-OsPIP2 markers displayed similar localization patterns (**Figures 3N, P, R**) as OsCTP1-GFP, Osγ-TIP-GFP, and GFP-OsPIP2, respectively. Unexpectedly, we were unable to obtain *DsRed-AtCCASP*-, *GFP-mTn*-, or *DsRed-OsH2B*-transgenic rice plants expressing obvious fluorescent signals (data not shown). Protoplasts isolated from the above transgenic rice lines were subjected to microscopic analysis, and the results were consistent with the observations in sheath cells (**Figure 4**).

To rule out the possibility that the *DsRed-AtCCASP*, *GFP-mTn*, and *DsRed-OsH2B* cassettes were not working for expression, we further transiently evaluated the three expression cassettes in *N. benthamiana* leaf cells and in rice protoplasts. Transient expression assays showed that the DsRed-AtCCASP, GFP-mTn, and DsRed-OsH2B fusions could be expressed in *N. benthamiana* leaves and rice protoplasts, producing fluorescent signals targeted to the Golgi apparatus, actin cytoskeleton, and nucleus, respectively (**Supplementary Figure S1**). These results indicated that the *DsRed-AtCCASP*, *GFP-mTn*, and *DsRed-OsH2B* cassettes were working for expression. Thus, it remains to be determined whether the three fusion proteins had harmful effects or there are other reasons that resulted in the failure to obtain transgenic rice lines stably expressing DsRed-AtCCASP, GFP-mTn, or DsRed-OsH2B.

### Organelle Marker Lines for Co-Localization Studies

**Figure 5:**
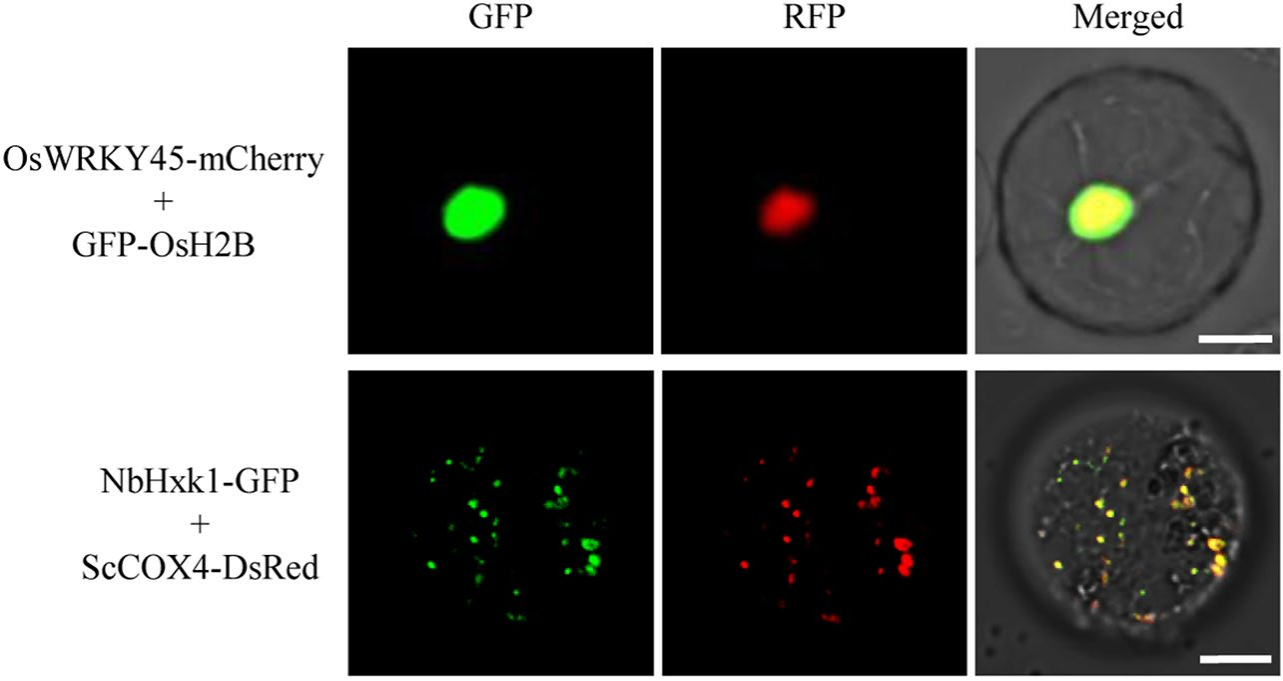
Co-localization analysis of OsWRKY45-mCherry and NbHxk1-green fluorescent protein (GFP) in protoplasts of the nuclear and mitochondrial marker lines. The *OsWRKY45-mCherry* and *NbHxk1-GFP* cassettes were transfected into protoplasts of the GFP-OsH2B (upper panel), and ScCOX4-DsRed (lower panel) rice lines, respectively. Scale bars, 10 μm.

To test the utility of the GFP/DsRed-fusion organelle marker lines for protein co-localization studies, we selected a rice transcriptional factor OsWRKY45 (Shimono et al., 2007) and a *N. benthamiana* mitochondria-associated hexokinase NbHxk1 (Kim et al., 2006) for proof-of-concept testing. OsWRKY45 fused with a mCherry and NbHxk1 fused with a GFP, were expressed in protoplasts of the GFP-OsH2B and ScCOX4-DsRed rice lines, respectively. The results showed that the red fluorescence of OsWRKY45-mCherry was merged with the nucleus GFP-OsH2B marker, and the fluorescent signal of NbHxk1-GFP was partly merged with the mitochondria ScCOX4-DsRed marker (**Figure 5**). Overall, these experiments demonstrated that using protoplasts of the organelle marker lines can facilitate protein co-localization studies in rice cells.

### Green Fluorescent Protein-OsH2B Marker Line for Detecting Translocation of *Magnaporthe oryzae* Effector Into Host Rice Cells

**Figure 6:**
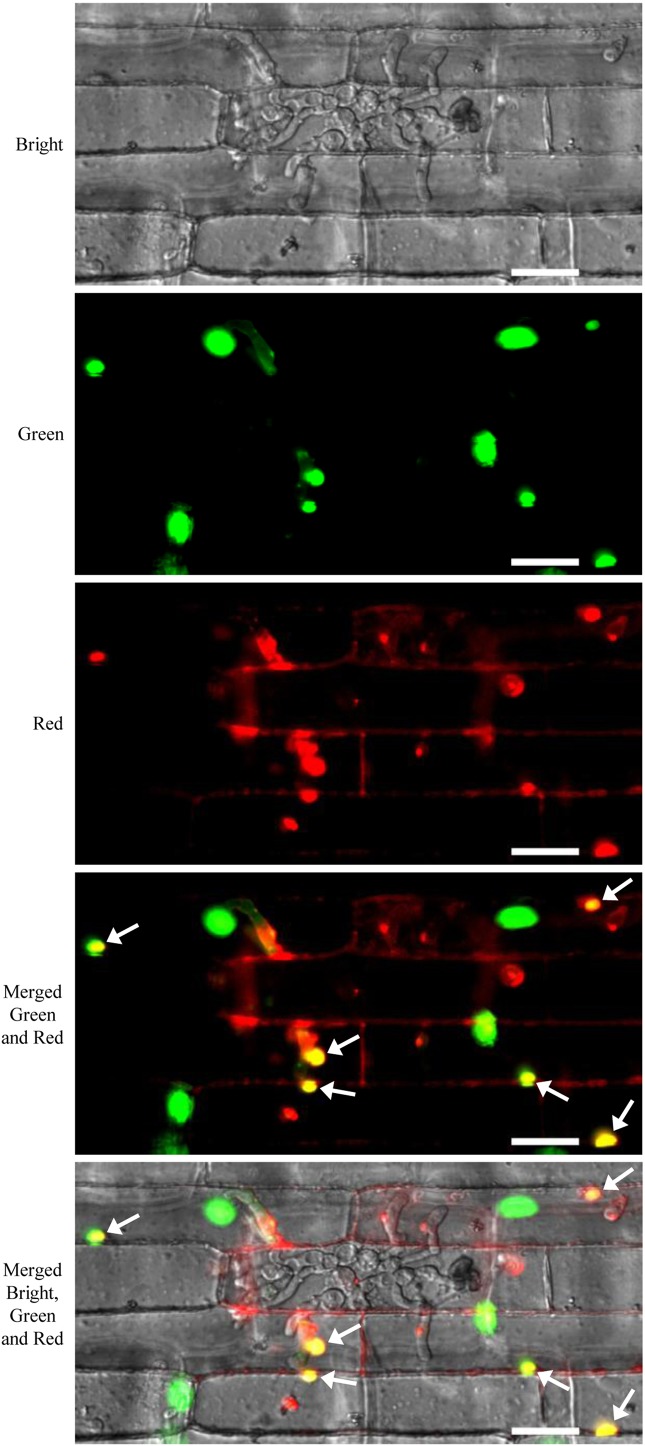
Live-cell imaging of *Magnaporthe oryzae* PWL2-mChery-NSL in rice cells of the green fluorescent protein (GFP)-OsH2B marker line. Rice leaf sheaths were inoculated with a transgenic *M. oryzae* isolate carrying a *PWL2:mCherry : NLS* expression cassette. Arrows indicate co-localization of PWL2-mCherry-NSL with the nuclear GFP-OsH2B marker. Scale bars, 25 μm.

FP-tagged organelle marker plants provided powerful tool to study dynamics of proteins or organelles in plant cells in response to various stimuli, including pathogen attack. For example, *Arabidopsis* plants expressing various GFP-tagged organelles have been successfully used for investigating the subcellular reorganization of host cells in response to pathogen attack (Takemoto et al., 2003; Koh et al., 2005; Lu et al., 2012). In this study, we performed sheath-inoculation of the GFP-OsH2B rice plants with a transgenic *M. oryzae* isolate expressing a PWL2 effector fused with mCherry and a nuclear localization signal (PWL2:mCherry : NLS) (Khang et al., 2010). Our result confirmed that PWL2:mCherry : NLS was translocated into rice cells, as indicated by co-localization with the nucleus GFP-OsH2B marker (**Figure 6**). This experiment also demonstrated that the organelle marker lines will be useful resources for studying dynamics of proteins or organelles in rice cells in the interactions between rice and pathogens.

## Discussion

In this study, we developed a set of plant expression cassettes for GFP- and DsRed-fusion organelle markers. The markers designed to target ER, mitochondria, Golgi apparatus, peroxisome, or actin cytoskeleton were based on fusion of GFP or DsRed with the well-established targeting proteins or motifs (Mathur, 2007), and the plastid, tonoplast, plasma membrane, and nucleus markers were constructed by fusing GFP or DsRed to rice orthologs of the OsCTP1 transit peptide, Osγ-TIP, OsPIP2, and OsH2B, respectively. The binary constructs of the organelle markers were used to generate stable transgenic rice plants. Microscopic analysis of sheath cells and protoplasts of transgenic rice plants showed that most of the GFP- or DsRed-fusions could be stably expressed in rice cells. In addition, we observed no significant difference in morphological traits between wild-type and the organelle marker lines (data not shown). However, we were unable to obtain transgenic plants expressing fluorescent signals of DsRed-AtCCASP, GFP-mTn, or DsRed-OsH2B, although the DsRed-AtCCASP, GFP-mTn, and DsRed-OsH2B fusions could be transiently expressed in *N. benthamiana* leaf epidermal cells and in rice protoplasts (**Supplementary Figure S1**). Indeed, we also generated another set of expression cassettes of *DsRed-AtCCASP*, *GFP-mTn*, and *DsRed-OsH2B*, in which the fusion organelle marker genes were driven by a strong maize ubiquitin-1 promoter (Christensen et al., 1992; Chen et al., 2009). Similarly, no transgenic plants expressing obvious fluorescent signals of DsRed-AtCCASP, GFP-mTn, or DsRed-OsH2B were obtained with multiple tries (data not shown). While the reason for failure to obtain the DsRed-AtCCASP, GFP-mTn, or DsRed-OsH2B marker lines remains to be investigated, in the future, we will try to develop rice marker lines expressing green fluorescence targeted to the actin cytoskeleton, and lines expressing red fluorescence targeted to the Golgi apparatus and nucleus, respectively, using mCherry (Shaner et al., 2004) or other FP variants as reporters.

The availability of organelle marker lines will facilitate protein subcellular localization studies. Both our results and previous report (Wu et al., 2016) demonstrated that protoplasts from the rice organelle marker lines were suitable for transfection with fusion constructs of the protein of interest with FPs, and the markers could be used as comparative standards in determining the subcellular localization of the protein of interest. The use of the transgenic organelle marker lines also has an advantage over transient expression assays in studying the dynamics of proteins or organelles in developmental or environmental contexts (Geldner et al., 2009), because the transgenic lines are able to provide consistent, intact tissues for investigation under complicated conditions. For example, the *Arabidopsis* organelle marker lines have been used to study the interactions of *Arabidopsis* with oomycete (Takemoto et al., 2003; Lu et al.,2012), and powdery mildew fungus (Koh et al., 2005). In the present study, we presented an example application of using the leaf sheaths of the GFP-OsH2B marker line for detecting translocation of a *M. oryzae* PWL2 effector into rice cells, demonstrating the feasibility of the rice organelle marker lines as useful tools for dissection of protein dynamics or protein reorganization involved in rice-pathogen interactions. In addition, we generated both GFP- and/or DsRed-tagged organelle markers lines. These lines will allow more flexible design of FP combinations for co-localization studies in rice.

In summary, we developed a set of rice lines stably expressing GFP- and/or DsRed-tagged organelle markers, and demonstrated the utility of the marker lines for protein subcellular localization studies, especially for studying dynamics of proteins in rice cells in the interaction between rice with blast fungus. These marker lines, along with previously established organelle markers and marker lines (Wu et al, 2016; Dangol et al., 2017), provide important resources for the rice research community.

## Data Availability Statement

The datasets generated for this study are available on request to the corresponding author.

## Author Contributions

ZW and SC conceived and designed the experiments. ZQC, WZ, LC, CL, TL, ZJC, HX, YH, LK, and XZ performed the experiments. ZQC, WZ, LC, CL, FW, and SC analyzed the data. ZQC, ZW, and SC wrote and revised the manuscript. All authors commented on the manuscript.

## Funding

This work was supported by grants from National Natural Science Foundation of China (U1405212, 31171808, 31601596) and grant from Fujian Provincial Science and Technology Program (2017R1019-1).

## Conflict of Interest

The authors declare that the research was conducted in the absence of any commercial or financial relationships that could be construed as a potential conflict of interest.
