# Supplementary material for: Green Fluorescent Protein- and Discosoma sp. Red Fluorescent Protein-Tagged Organelle Marker Lines for Protein Subcellular Localization in Rice
Source: Front Plant Sci. 2019 Nov 5;10:1421. doi: 10.3389/fpls.2019.01421 (PMC6848374; doi:10.3389/fpls.2019.01421)
Supplement: Supplementary file 1 [file Table_1.doc]

**Supplementary Table S1.** Primers used in this study

| **No.** | **Primers** | **Sequence: 5′- 3′** | **Comments** |
| --- | --- | --- | --- |
| 1 | GFP-F | ATGGTGAGCAAGGGCGAGGA | Primers to amplify *GFP* |
| 2 | GFP-R | CATGGACGAGCTGTACAAGTAA |
| 3 | AtWAK2-G-F1 | ATGAAGGTACAGGAGGGTTTG | Primers to amplify *AtWAK2-GFP-HDEL* |
| 4 | AtWAK2-G-R1 | GCCCTTGCTCACCATAGGTTGCCCCTTGA |
| 5 | AtWAK2-G-F2 | TCAAGGGGCAACCTATGGTGAGCAAG |
| 6 | AtWAK2-G-R2 | TTAAAGCTCATCATGCTTGTACAGC |
| 7 | CoxIV-G-F1 | ATGTTGTCACTACGTCAATCTATAAG | Primers to amplify *COX4-GFP* |
| 8 | CoxIV-G-R1 | CCCTTGCTCACCATGGGTTTTTGCTGAA |
| 9 | CoxIV-G-F2 | CAGCAAAAAACCCATGGTGAGCAAGG |
| 10 | CoxIV-G-R2 | TTACTTGTACAGCTCGTCCATG |
| 11 | G-AtCCASP-F1 | ATGGTGAGCAAGGGCGAGGA | Primers to amplify *GFP-AtCCASP* |
| 12 | G-AtCCASP-R1 | ATCATGGTTATAGTCCTTGTACAGCTCGTC |
| 13 | G-AtCCASP-F2 | CGAGCTGTACAAGGACTATAACCATGA |
| 14 | G-AtCCASP-R2 | TTAAAGACCGTGAGGAAGG |
| 15 | G-PTS1-F | ATGGTGAGCAAGGGCGAGGA | Primers to amplify *GFP-PTS1* |
| 16 | G-PTS1-R | TTACAGCTTCGACTTGTACAGCTCGTC |
| 17 | G-mTn-F1 | ATGGTGAGCAAGGGCGAGGA | Primers to amplify *GFP-mTalin* |
| 18 | G-mTn-R1 | GCAGCTTCTAGGATCTTGTACAGCTCGTC |
| 19 | G-mTn-F2 | GCTGTACAAGATCCTAGAAGCTGCCAA |
| 20 | G-mTn-R2 | TTAGTGCTCGTCTCGAAGCTCTGAA |
| 21 | CTP1-G-F1 | ATGGCTGCTGCCTTCTCC | Primers to amplify *OsCTP1-GFP* |
| 22 | CTP1-G-R1 | TCGCCCTTGCTCACCATGGCCATCACCTTGAAC |
| 23 | CTP1-G-F2 | TTCAAGGTGATGGCCATGGTGAGCAAGGGCGA |
| 24 | CTP1-G-R2 | TTACTTGTACAGCTCGTCCATG |
| 25 | TIP-G-F1 | ATGCCGATCCGCAATATCGC | Primers to amplify *OsTIP-GFP* |
| 26 | TIP-G-R1 | CTTGCTCACCATGTAGTCGGTGGTGGGGA |
| 27 | TIP-G-F2 | ACCACCGACTACATGGTGAGCAAGG |
| 28 | TIP-G-R2 | TTACTTGTACAGCTCGTCCATG |
| 29 | G-P1P2-F1 | ATGGTGAGCAAGGGCGAGGA | Primers to amplify *GFP-OsPIP2* |
| 30 | G-P1P2-R1 | ATGTCTTTCGCCATCTTGTACAGCTCGTC |
| 31 | G-P1P2-F2 | AGCTGTACAAGATGGCGAAAGACA |
| 32 | G-P1P2-R2 | TCAGGCGTTGCTCCGGTAG |
| 33 | G-H2B-F1 | ATGGTGAGCAAGGGCGAGGA | Primers to amplify *GFP-OsH2B* |
| 34 | G-H2B-R1 | CTCCGCCTTGGGCGCCATCTTGTACAGCTCGTC |
| 35 | G-H2B-F2 | GACGAGCTGTACAAGATGGCGCCCAAGGCGGAG |
| 36 | G-H2B-R2 | TTAAGACGACGTGAACTTGGTGACG |
| 37 | DsRed-F | ATGGCCTCCTCCGAGAAC | Primers to amplify *DsRed* |
| 38 | DsRed-R | TTATCTAGATCCGGTGGATC |
| 39 | AtWAK2-R-F1 | ATGAAGGTACAGGAGGGTTTG | Primers to amplify *AtWAK2-DsRed-HDEL* |
| 40 | AtWAK2-R-R1 | CGGAGGAGGCCATAGGTTGCCCCTTG |
| 41 | AtWAK2-R-F2 | GTCAAGGGGCAACCTATGGCCTCCTCCGA |
| 42 | AtWAK2-R-R2 | TTAAAGCTCATCATGTCTAGATCCGGTGGA |
| 43 | CoxIV-R-F1 | ATGTTGTCACTACGTCAATCTATAAG | Primers to amplify *COX4-DsRed* |
| 44 | CoxIV-R-R1 | GAGGAGGCCATGGGTTTTTGCTGAA |
| 45 | CoxIV-R-F2 | CTTCAGCAAAAACCCATGGCCTCCTCCGA |
| 46 | CoxIV-R-R2 | TTATCTAGATCCGGTGGATC |
| 47 | R-AtCCASP-F1 | ATGGCCTCCTCCGAGAAC | Primers to amplify *DsRed-AtCCASP* |
| 48 | R-AtCCASP-R1 | ATCATGGTTATAGTCTCTAGATCCGGTG |
| 49 | R-AtCCASP-F2 | CCACCGGATCTAGAGACTATAACCATGA |
| 50 | R-AtCCASP-R2 | TTAAAGACCGTGAGGAAGG |
| 51 | R-PTS1-F | ATGGCCTCCTCCGAGAAC | Primers to amplify *DsRed-PTS1* |
| 52 | R-PTS1-R | TTACAGCTTCGATCTAGATCCGGTG |
| 53 | R-mTn-F1 | ATGGCCTCCTCCGAGAAC | Primers to amplify *DsRed-mTalin* |
| 54 | R-mTn-R1 | AGCTTCTAGGATTCTAGATCCGGTG |
| 55 | R-mTn-F2 | ACCGGATCTAGAATCCTAGAAGCTGCCA |
| 56 | R-mTn-R2 | TTAGTGCTCGTCTCGAAGCTCTGAA |
| 57 | CTP1-R-F1 | ATGGCTGCTGCCTTCTCC | Primers to amplify *OsCTP1-DsRed* |
| 58 | CTP1-R-R1 | CGGAGGAGGCCATGGCCATCACCTTGA |
| 59 | CTP1-R-F2 | GTTCAAGGTGATGGCCATGGCCTCCTCCGA |
| 60 | CTP1-R-R2 | TTATCTAGATCCGGTGGATC |
| 61 | TIP-R-F1 | ATGCCGATCCGCAATATCGC | Primers to amplify *OsTIP-DsRed* |
| 62 | TIP-R-R1 | TCTCGGAGGAGGCCATGTAGTCGGTGGTG |
| 63 | TIP-R-F2 | TCCCCACCACCGACTACATGGCCTCCTCCGA |
| 64 | TIP-R-R2 | TTATCTAGATCCGGTGGATCCCG |
| 65 | R-P1P2-F1 | ATGGCCTCCTCCGAGAAC | Primers to amplify *DsRed-OsPIP2* |
| 66 | R-P1P2-R1 | ATGTCTTTCGCCATTCTAGATCCGGTGG |
| 67 | R-P1P2-F2 | ACCGGATCTAGAATGGCGAAAGACATTG |
| 68 | R-P1P2-R2 | TCAGGCGTTGCTCCGGTAG |
| 69 | R-H2B-F1 | ATGGCCTCCTCCGAGAAC | Primers to amplify *DsRed-OsH2B* |
| 70 | R-H2B-R1 | CCGCCTTGGGCGCCATTCTAGATCCGGTGGA |
| 71 | R-H2B-F2 | TCCACCGGATCTAGAATGGCGCCCAAGGCGG |
| 72 | R-H2B-R2 | TTAAGACGACGTGAACTTGGTGACG |
| 73 | 45-mCherry-F1 | ATGACGTCATCGATGTCGCC | Primers to amplify *OsWRKY45-mCherry* |
| 74 | 45-mCherry-R1 | CCCTTGCTCACCATAAAGCTCAAACCCATAA |
| 75 | 45-mCherry-F2 | ATGGTGAGCAAGGGCGAGGA |
| 76 | 45-mCherry-R2 | TCACTTGTACAGCTCGTCCA |
| 77 | HxK-GFP-F1 | ATGAAGAAAGCGACGGTGG | Primers to amplify *NbHxk1-GFP* |
| 78 | HxK-GFP-R1 | CCCTTGCTCACCATGGACTTATCTTCAAGGT |
| 79 | HxK-GFP-F2 | ACCTTGAAGATAAGTCCATGGTGAGCAAGGG |
| 80 | HxK-GFP-R2 | TTACTTGTACAGCTCGTCCA |
