# Supplementary figures and images for: Green Fluorescent Protein- and Discosoma sp. Red Fluorescent Protein-Tagged Organelle Marker Lines for Protein Subcellular Localization in Rice
Source: Front Plant Sci. 2019 Nov 5;10:1421. doi: 10.3389/fpls.2019.01421 (PMC6848374; doi:10.3389/fpls.2019.01421)

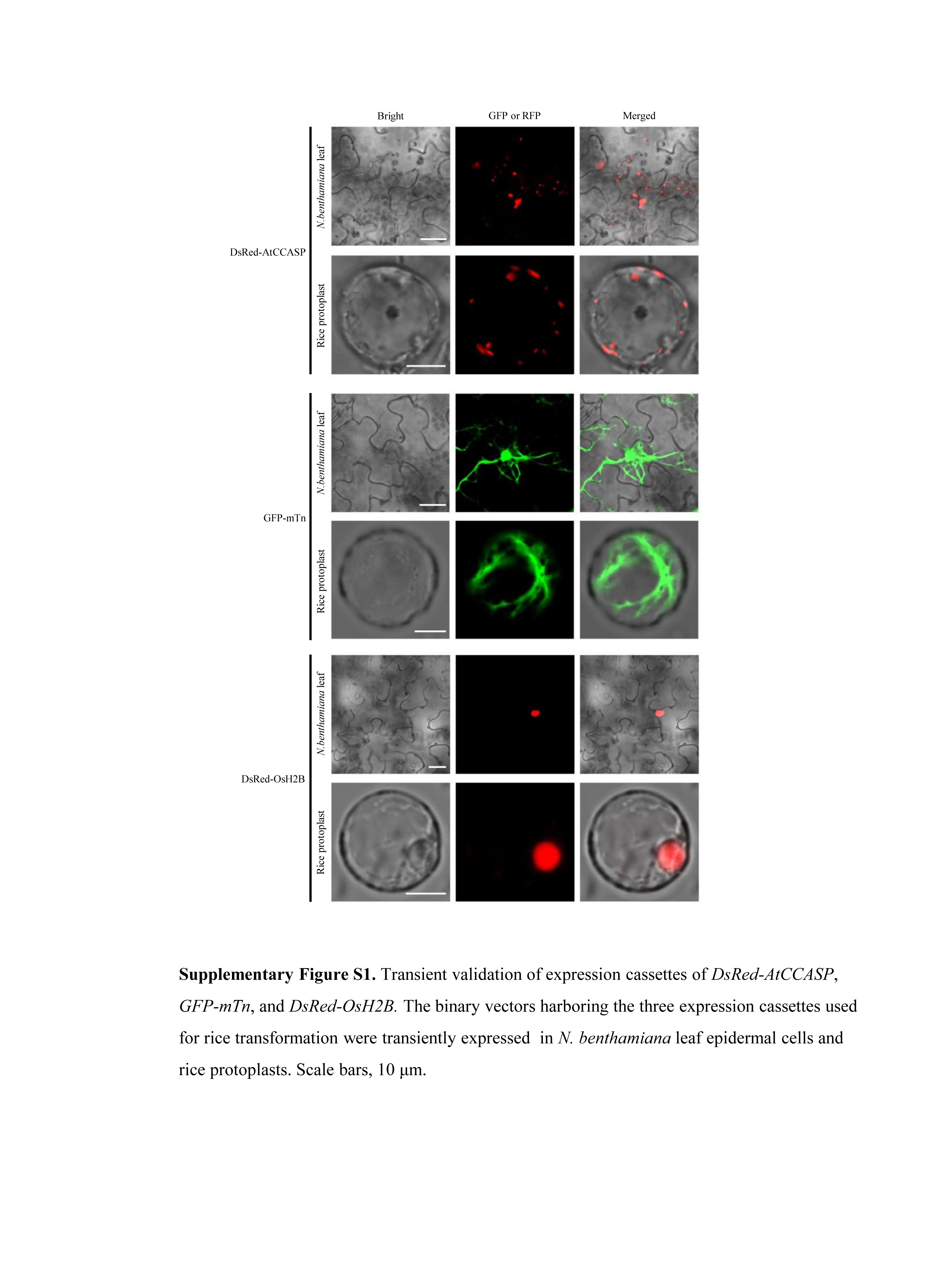

Supplement: Supplementary file 2 [file Image_1.jpeg]
